# New Voices in Virology: our inaugural cohort

**DOI:** 10.1128/jvi.00334-26

**Published:** 2026-03-30

**Authors:** Herman W. Favoreel, David E. Levy, Suchetana Mukhopadhyay

**Affiliations:** 1 Department of Translational Physiology, Infectiology and Public Health, Faculty of Veterinary Medicine, Ghent University 26656, Merelbeke, Belgium; 2 Vilcek Institute of Graduate Biomedical Sciences, NYU Grossman School of Medicine, NYU Langone Health 12297, New York, New York, USA; 3 Department of Biology, Indiana University 1772, Bloomington, Indiana, USA

## EDITORIAL

This year marks the 230th anniversary of Edward Jenner’s landmark smallpox vaccination of 8-year-old James Phipps on 14 May 1796, using material derived from cowpox lesions. Nearly a century later, the conceptual foundations of virology began to emerge through the work of Dmitri Ivanovsky, Martinus Beijerinck, Friedrich Loeffler, and Paul Frosch, who demonstrated the existence of infectious agents capable of passing through bacteria-retaining filters and invisible to light microscopy. More than 4 decades later, upon the development of transmission electron microscopy by Ernst Ruska and Max Knoll, virus particles could be visualized for the very first time in 1939. More than half a century ago, David Baltimore introduced a systematic classification scheme for viruses based on their nucleic acid content and mode of replication, setting the stage for the modern era of molecular virology. From these beginnings, virology has expanded into a broad and dynamic discipline spanning structural biology, molecular genetics, immunology, ecology, evolution, and translational science.

Viruses remain formidable threats to human, animal, and plant health. At the same time, fundamental discoveries in virology have catalyzed transformative advances in biomedical science, leading to highly effective vaccines and antiviral therapies against some of the most consequential and pandemic viral diseases. Moreover, viral evolution has produced extraordinarily efficient systems for delivering genetic material into host cells—mechanisms now harnessed for gene therapy and other therapeutic applications. There is no ambiguity about the vitality and impact of virology research, as it is thriving, innovative, and central to modern biology and medicine.

In this spirit, we are pleased to highlight the inaugural cohort of New Voices in Virology (NViV), an initiative of the American Society for Microbiology’s *Journal of Virology*. From more than 250 applicants, 41 outstanding early-career investigators were selected to author minireviews on topics of their choosing and passion. The scope of these contributions reflects the remarkable breadth and intellectual energy of contemporary virology.

This editorial accompanies the first set of NViV minireviews published. We hope readers will find them as stimulating and forward-looking as we do. Additional NViV minireviews will appear in the months ahead. We encourage emerging virologists to apply for the next NViV cycle, which will open in June 2026, as we continue to foster and showcase the next generation of leaders in our field.

